# An audit and feedback strategy does not improve compliance with surgical antimicrobial prophylaxis guidelines

**DOI:** 10.1186/2047-2994-4-S1-P82

**Published:** 2015-06-16

**Authors:** V Vitrat, A Jean, J Fiot, C Janssen, S Nguyen, F Guerin, L Pagani

**Affiliations:** 1 Antimicrobial Management Team, Annecy-Genevois Hospital Centre, Annecy, France; 2 Infectious Diseases Unit, Bolzano Central Hospital, Bolzano, Italy

## Introduction

The efficacy of surgical antibiotic prophylaxis (SAP) in reducing surgical site infections has been clearly ascertained, provided that the patient receives the right antibiotic, at the right dose, at the right time before surgical incision.

## Objectives

This study was carried out to assess whether an evaluation of routine SAP practices may improve subsequent compliance with SAP guidelines (GL) through surgeon and anesthesiologist feedback.

## Methods

In 2013, we carried out an audit on SAP in our 850-bed tertiary hospital in France, at two scheduled time points (T-1 and T-2). We rated a point-by-point compliance with national GL indicators (SAP indication/no indication, type of antibiotic, dose, time of injection before incision, duration) for all surgical interventions recorded in those given days. We then presented the results to surgeons and anesthesiologists, focusing on negative performance measures and providing support to fill the gaps. One year after T-1, we performed an identical analysis (T-3) to evaluate the impact of the strategy on daily practice.

## Results

124 surgical interventions were recorded at T-1 and T-2, and 56 at T-3. The overall rate of conformity with GL was 60% for T-1 and T-2, and 54% for T-3. Two out of 124 procedures (1.6%) did not receive SAP at T-1 and T-2, and 1 out of 56 at T-3 (1.8%), despite indication. SAP was rarely anticipated during pre-operative anesthesiology consultation, whereas allergies and comorbidities were always detailed. The drug and the dose were correctly administered in all the interventions. The number of reinjections was also correct according to the duration of each specific procedure. However, the timing of first injection was too close to incision (< 30 minutes), with conformity rate as low as 30% in T-1 and T-2, and 14% in T-3. SAP duration was < 48h in 100% of the cases.

## Conclusion

This audit and feedback strategy failed to improve compliance with SAP GL indicators in the post-feedback period. However, the lower number of procedures recorded in the T-3 day may have affected the overall performance of the system. Alternative means aimed at improving quality of care and reducing SAP practice variability within our surgical department should be carried out.

## Disclosure of interest

None declared.

